# Toxicity of the herbicides diuron, propazine, tebuthiuron, and haloxyfop to the diatom *Chaetoceros muelleri*

**DOI:** 10.1038/s41598-020-76363-0

**Published:** 2020-11-11

**Authors:** Marie C. Thomas, Florita Flores, Sarit Kaserzon, Timothy A. Reeks, Andrew P. Negri

**Affiliations:** 1 Australian Institute of Marine Science, Townsville, QLD 4810 Australia; 2 Queensland Alliance for Environmental Health Sciences (QAEHS), The University of Queensland, Woolloongabba, QLD 4102 Australia

**Keywords:** Microbial ecology, Environmental impact, Marine biology, Environmental sciences

## Abstract

Conventional photosystem II (PSII) herbicides applied in agriculture can pose significant environmental risks to aquatic environments. In response to the frequent detection of these herbicides in the Great Barrier Reef (GBR) catchment area, transitions towards ‘alternative’ herbicides are now widely supported. However, water quality guideline values (WQGVs) for alternative herbicides are lacking and their potential ecological impacts on tropical marine species are generally unknown. To improve our understanding of the risks posed by some of these alternative herbicides on marine species under tropical conditions, we tested the effects of four herbicides on the widely distributed diatom *Chaetoceros muelleri.* The PSII herbicides diuron, propazine, and tebuthiuron induced substantial reductions in both 24 h effective quantum yields (ΔF/F_m_′) and 3-day specific growth rates (SGR). The effect concentrations, which reduced ΔF/F_m_′ by 50% (EC_50_), ranged from 4.25 µg L^−1^ diuron to 48.6 µg L^−1^ propazine, while the EC_50_s for SGR were on average threefold higher, ranging from 12.4 µg L^−1^ diuron to 187 µg L^−1^ tebuthiuron. Our results clearly demonstrated that inhibition of ΔF/F_m_′ in PSII is directly linked to reduced growth (R^2^ = 0.95) in this species, further supporting application of ΔF/F_m_′ inhibition as a valid bioindicator of ecological relevance for PSII herbicides that could contribute to deriving future WQGVs. In contrast, SGR and ΔF/F_m_′ of *C. muelleri* were nonresponsive to the non-PSII herbicide haloxyfop at the highest concentration tested (4570 µg L^−1^), suggesting haloxyfop does not pose a risk to *C. muelleri*. The toxicity thresholds (e.g. no effect concentrations; NECs) identified in this study will contribute to the derivation of high-reliability marine WQGVs for some alternative herbicides detected in GBR waters and support future assessments of the cumulative risks of complex herbicide mixtures commonly detected in coastal waters.

## Introduction

### Herbicide contamination in the Great Barrier Reef

Herbicide contamination of nearshore waters is common across tropical regions, including the Caribbean^1^, Mexico^2^, Central America^3^ and the Asia-Pacific^4–8^; however, the Great Barrier Reef (GBR) World Heritage Area located on the east coast of northern Queensland, Australia represents the most studied location for herbicide contamination in tropical waters^9^. The GBR is the world’s largest reef ecosystem, containing extensive areas of seagrass meadows, mangroves, and coral reefs^10^. The GBR catchment (> 400,000 km^2^) accommodates a large agricultural industry that comprises of row crops (mainly sugarcane cultivation and horticulture) and cattle grazing in which pesticides are commonly applied to control weeds and other pests^9,11,12^. With > 35 major rivers discharging into the GBR lagoon, pesticide contamination from nearshore agricultural runoff is recognized as one of several threats faced by tropical nearshore ecosystems that need to be managed to maintain the health of this ecosystem^13^. Pesticides detected in waters of the GBR include herbicides, insecticides and fungicides, with long-term water quality monitoring programs most frequently detecting a group of five photosystem II (PSII) herbicides (diuron, ametryn, atrazine, tebuthiuron, and hexazinone)^9,14–17^. PSII herbicides are designed to target weeds by competing with plastoquinone for the secondary quinone Q_B_ binding site on the D_1_ protein within the thylakoid membrane^18^. This results in the interruption of the electron transport from the primary quinone Q_A_ to Q_B_ and subsequently, light-induced degradation of the D_1_ protein and reduced photochemical energy conversion within PSII^18^. Since all plants rely on the function of PSII, these herbicides can be equally effective at harming non-target species as the weeds they were designed to control. Due to their widespread application in agricultural industries, these herbicides are found throughout the nearshore waters of the GBR, but more frequently and at higher concentrations following riverine flood events (December to April)^12,19–21^ in which peak concentrations of up to 22 µg L^−1^ diuron have been detected in grab samples flowing into the GBR lagoon^12^. However, their continuous application and persistence^22,23^ contributes to year-round detections^15,24^. Consequently, these herbicides are considered ‘priority’ herbicides for management action designed to reduce the potential impacts of contaminants in waters of the GBR and its catchments^13^. To help achieve targeted reductions in priority herbicide loads, ’alternative’ PSII herbicides and ‘alternative’ non-PSII herbicides are increasingly applied as substitutes for effective weed control^25^. At present, sixteen alternative herbicides with five modes of action have been detected in GBR waters in addition to the priority PSII herbicides^24^. Alternative herbicides can exhibit similarities in physico-chemical properties to the priority PSII herbicides and in some cases, can be just as toxic to non-target marine species^26^. Nevertheless, their potential ecological impacts, particularly of non-PSII herbicides, on aquatic environments are generally unknown^9^.

### Improving water quality guideline values for pesticides

The risks posed to aquatic habitats by contaminants are generally assessed by comparing measured concentrations in the field against water quality guideline values (WQGVs). In Australia, national WQGVs (referred to by the Australian and New Zealand Guidelines for Fresh and Marine Water Quality (ANZG)^27^ as default GVs) are derived to protect 99%, 95%, 90% and 80% (PC99, 95, 90, 80, respectively) of marine and freshwater species by assessing community sensitivities from species sensitivity distributions (SSDs)^28^. These SSDs are derived from toxicity threshold data of at least five species from at least four phyla that are representative of the receiving environment^28^. Currently, the ANZG include marine WQGVs for only four alternative herbicides: bromacil, simazine, 2,4-D, and MCPA, and the priority PSII herbicides (except for ametryn)^27^. However, with exception from diuron, these marine WQGVs are of low reliability as they were adapted from freshwater toxicity thresholds^27^.

Regular monitoring of pesticides in the GBR has found that exceedances of WQGVs by individual herbicides in the GBR marine waters occur only occasionally^15,16,24^; however, approximately 80% of the water samples collected in the GBR catchment area between 2011 and 2015 contained mixtures of up to 20 pesticides with two to four modes of action^29^. Consequently, there is a strong likelihood of additivity or synergistic interactions between multiple herbicides, and the total toxicity of herbicide mixtures should be considered in monitoring programs and for risk assessments^14,30–32^. To predict the cumulative risk of herbicide mixtures, a more comprehensive risk assessment approach has been proposed which applies the multisubstance-potentially affected fraction (ms-PAF) method^33^. In cases where the combined concentrations of multiple co-occurring herbicides are considered using the ms-PAF approach, WQGV exceedances in the GBR become more frequent^24^. The ms-PAF method has also recently been extended to adjust herbicide WQGVs for heatwave conditions often faced by tropical marine species^34^. Improved WQGVs for alternative herbicides are therefore required so that the ms-PAF method can take into consideration all herbicides detected in water samples for assessing the total risk. A revision of the current WQGVs, including 13 alternative herbicides, has been proposed based on all available marine and freshwater toxicity data^35,36^. Nevertheless, most of the proposed guideline values (PGVs) are still of low reliability and many data gaps remain, especially for marine species. Consequently, additional toxicity testing of most herbicides using marine phototrophs is recommended for improving their reliability^35,36^.

### Toxicity testing with marine microalgae

Marine microalgae form an essential functional group as primary producers. However, herbicide-induced damage to PSII leads to declining growth rates and biomass of microalgae and consequently, may initiate indirect bottom-up effects on higher trophic levels due to changes in their community structure^37^. Non-PSII herbicides can also affect microalga, but their vulnerability depends on whether the mode of action of the herbicide is also relevant to each specific type of algae. Their ecological importance, potential vulnerability to herbicides, along with rapid growth rates that allow for chronic exposure testing in a short period, mean that marine microalgae represent a suitable taxon to contribute to improving WQGVs. Currently, SSDs used to derive high-quality WQGVs require ecologically relevant toxicity data, and for microalgae, the inhibition of growth is the most common endpoint^27,28^. Another more rapid and sensitive technique to quantify the toxicity of PSII herbicides to marine phototrophs takes advantage of increased chlorophyll *a* fluorescence emissions that result from the excess excitation energy that would normally drive electron transport in PSII but is blocked by these herbicides^38^. This results in reduced photosynthetic efficiency (effective quantum yield: ΔF/F_m_′) which can be measured by pulse amplitude modulation (PAM) fluorometry^39^. PAM fluorometry has been extensively applied for assessing sub-lethal effects of PSII herbicides in microalgae^40–43^; however, several studies have demonstrated that this method can be far less sensitive to non-PSII herbicides, where the mode of action does not involve PSII^26,44,45^. Nevertheless, further assessment of ΔF/F_m_′ inhibition as an effective endpoint for herbicides is warranted to investigate its suitability as an ecologically relevant endpoint to support herbicide risk assessments.

In order to improve WQGVs for herbicides detected in GBR waters, more toxicity data is required for deriving high-quality SSDs. Here, we tested the individual effects of four herbicides on the growth and ΔF/F_m_′ of the diatom *Chaetoceros muelleri*, which was selected as a representative of the phylum Bacillariophyta, generally underrepresented in current SSDs. Additionally, this study aimed to estimate no effect concentrations (NECs) for single herbicides which are the preferred toxicity thresholds for inclusion in SSDs to derive WQGVs^28^. Based on consultation with the Water Quality and Investigation Team at the Queensland Department of Environment and Science (DES) three herbicides that indicated current data gaps were chosen for testing, along with the reference PSII herbicide diuron. The tested herbicides included the PSII herbicides tebuthiuron and propazine, as well as the non-PSII herbicide haloxyfop. The toxicity thresholds identified here provide valuable toxicity data for some PSII and a non-PSII herbicide detected in GBR waters to contribute to improved WQGVs that are necessary for adequate protection of marine species and application in risk assessments.

## Results

### Toxicity test performance

Control growth rates of the test species *C. muelleri* were consistently > 1 doublings day^-1^ across all four 3-day experiments with SGR ranging from 1.41 ± 0.05 day^−1^ to 1.68 ± 0.05 day^−1^ (mean ± SD) (Table 1). The percent coefficient of variation (% CV) for each test was ≤ 5%, indicating test acceptability^46^ for all toxicity tests (Table 1). Chlorophyll fluorescence control measurements were also consistent over the exposure period of 24 h, with ΔF/F_m_′ control measurements across all tests varying between 0.418 ± 0.015 and 0.478 ± 0.005 (mean ± SD). The ethanol carrier solvent (< 0.01% v/v) had no significant influence on SGR compared with filtered seawater (FSW) after 3-days (ANOVA, F_ethanol_ (1,3) = 3.23, p = 0.17). The effect of the reference toxicant diuron applied at 4 µg L^−1^ across all experiments also inhibited SGR and ΔF/F_m_′ consistently across all tests (Table 1). Physicochemical measurements of salinity (33.3–35.3 psu; range across all herbicide tests), dissolved oxygen (8.0–8.4 mg L^−1^), and temperature (26.9–27.8 °C) indicated little variation within each treatment and across all tests (Table S-3). Changes in pH across all tests varied between 8.0 and 8.5 over 3-days and remained within the acceptable range of < 1 pH unit change for each test (Table S-3)^46^. Nominal and measured concentrations of each herbicide are presented in Table S-2.

**Table 1:** Assay performance summary.

Herbicide	pH range^a^	Specific growth rate (SGR day^−1^)		Ref. (%)	Photosynthetic efficiency (ΔF/F_m_′)		Ref. (%)
		SWC	CV (%)		SWC	CV (%)	
Diuron	8.17–8.52	1.68 ± 0.05	3	21.0 ± 2.79	0.451 ± 0.007	2	44.8 ± 0.75
Propazine	8.16–8.39	1.49 ± 0.07	5	24.1 ± 1.42	0.478 ± 0.005	1	41.0 ± 3.19
Tebuthiuron	8.00–8.41	1.56 ± 0.03	2	25.4 ± 2.45	0.468 ± 0.007	2	39.2 ± 0.53
Haloxyfop	7.99–8.24	1.41 ± 0.05	3	21.1 ± 1.57	0.418 ± 0.02	5	56.5 ± 4.86

Seawater pH range, seawater control (SWC) measurements of specific growth rate (SGR day^−1^) and photosynthetic efficiency (ΔF/F_m_′), percent coefficient of variation (% CV) and reference diuron (4 µg L^−1^) percent inhibition effect (Ref. (%)) of each herbicide test (mean ± SD; n = 5 per treatment). All physicochemical measurement data can be found in Table S-3.

^a^Range of pH from 0–72 h across treatments (Table S-3).

### Toxicity of herbicides to microalgae

**Figure 1:**
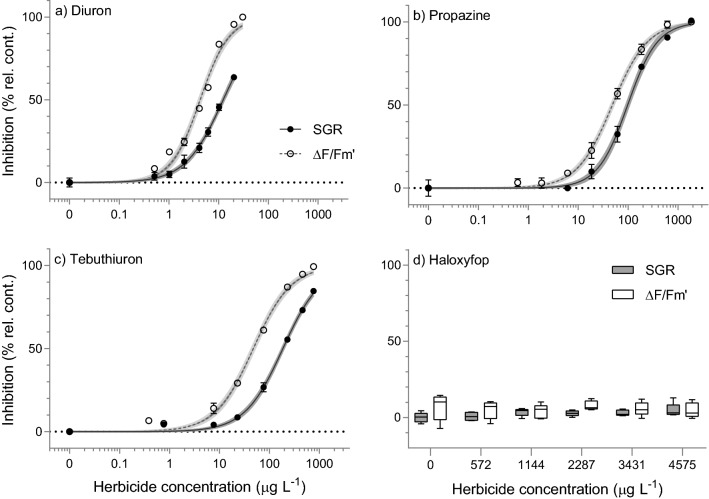
Concentration–response curves for EC_x_ derivation. Sigmoidal, 4-parameter curve fit and 95% confidence intervals (shaded area) on the relative percent inhibition of 3-day specific growth rate (SGR) and 24 h effective quantum yield (ΔF/F_m_′) of *Chaetoceros muelleri* (mean ± SD) following herbicide exposure to (**a**) Diuron; (**b**) Propazine; (**c**) Tebuthiuron and boxplot showing inhibition of 3-day specific growth rate (SGR) and effective quantum yield (ΔF/F_m_′) in response to (**d**) Haloxyfop. All concentrations in µg L^−1^ (n = 5 for each treatment, error bars not visible are smaller than symbol).

**Figure 2:**
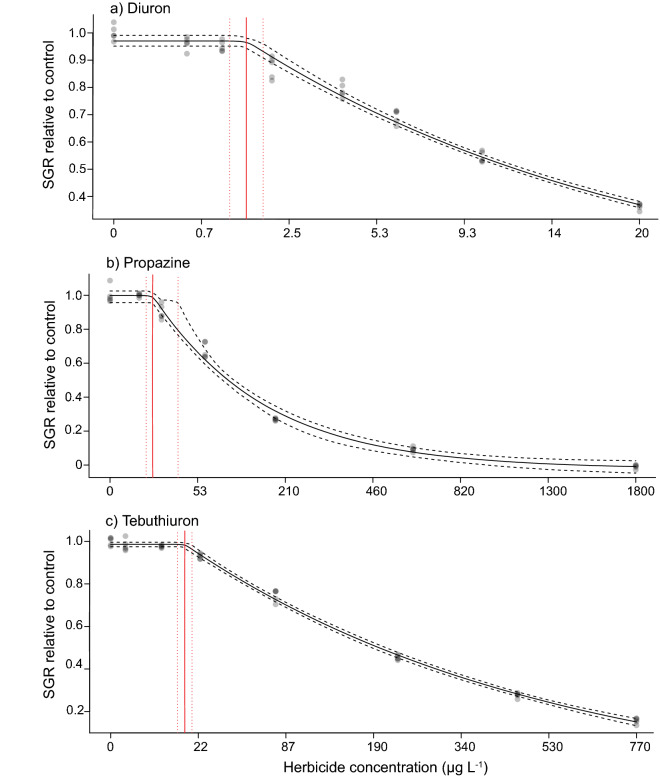
Concentration–response curves for NEC derivation. Bayesian non-linear gaussian model fit on the proportional decline in 3-day specific growth rate (SGR) relative to the control treatment (solid black line) and 95% confidence intervals (black dashed line) to derive the no effect concentration (NEC) (red line) and 95% confidence interval (red dashed line) of (**a**) Diuron; (**b**) Propazine; (**c**) Tebuthiuron. All concentrations in µg L^−1^.

Concentration-dependent inhibition of growth and photosynthetic activity (ΔF/F_m_′) was observed for all PSII herbicides tested (Fig. 1). These herbicides exhibited a broad range of potencies with diuron being the most toxic, inhibiting 50% of SGR and ΔF/F_m_′ (EC_50_) at 12.4 µg L^−1^ and 4.25 µg L^−1^, respectively (Table 2). A comparison of relative potencies (ReP, based on EC_50_ values) against the reference herbicide diuron revealed that the least potent PSII herbicide to SGR was tebuthiuron (ReP = 0.066), indicating 15-times lower toxicity than diuron (Rep = 1) (Table 2). Based on ReP values for ΔF/F_m_′ inhibition, the PSII herbicide propazine (ReP = 0.087) was least toxic to *C. muelleri* (Table 2) and was 11-fold less toxic than diuron (Table 2). The concentration–response curves all exhibited similar shapes and slopes (Fig. 1) with R^2^ values ≥ 0.98. The EC_10_ and predicted NEC values (from Figs. 1 and 2, respectively) were also reported in Table 2 and showed similar orders of toxicity. In contrast to the PSII herbicides, SGR and ΔF/F_m_′ of *C. muelleri* were not affected by the acetyl-CoA carboxylase (ACCase) inhibitor haloxyfop at the maximum concentration of 4570 µg L^−1^ tested and no significant differences between treatments by ANOVA (F (6,28) = 2.2, p = 0.07; F (5,28) = 1.5, p = 0.24, respectively) were detected (Fig. 1). Higher concentrations were not tested due to its low water solubility^47^.

**Table 2:** Toxicity threshold summary.

Herbicide	Endpoint	Specific growth rate (SGR)	Photosynthetic efficiency (ΔF/F_m_′)	SGR (EC_50_) : ΔF/F_m_′ (EC_50_)
Diuron	EC_50_	12.4 (11.8–13.0)	4.25 (3.96–4.55)	2.92
	EC_10_	1.79 (1.60–1.98)	0.97 (0.81–1.15)	
	NEC	1.47 (1.15–1.83)		
	*ReP*	*1*	*1*	
Propazine	EC_50_	98.2 (91.7–105)	48.6 (45.6–51.7)	2.02
	EC_10_	21.5 (18.4–25.0)	8.12 (7.04–9.33)	
	NEC	12.9 (9.29–32.0)		
	*ReP*	*0.126*	*0.087*	
Tebuthiuron	EC_50_	187 (179–195)	47.7 (44.1–51.5)	3.92
	EC_10_	26.8 (23.9–29.9)	6.95 (5.79–8.27)	
	NEC	16.0 (13.0–19.1)		
	*ReP*	*0.066*	*0.089*	
Haloxyfop	EC_50_	> 4570	> 4570	NA
	EC_10_	> 4570	> 4570	
	NEC	> 4570	> 4570	
	*ReP*	*NA*	*NA*	

Effect concentrations that inhibit the specific growth rate (SGR) and photosynthetic efficiency (ΔF/F_m_′) by 10% or 50% (EC_10_ and EC_50_ from Fig. 1) and no effect concentrations (NECs from Fig. 2), with 95% confidence intervals derived for Diuron, Propazine, Tebuthiuron and Haloxyfop. The potencies for each of the herbicides were contrasted using the relative equivalent potencies (ReP) in comparison to the reference herbicide diuron. NA indicates values could not be calculated. Concentrations are reported in µg L^−1^.

### Relationship between inhibition of effective quantum yield and growth

**Figure 3:**
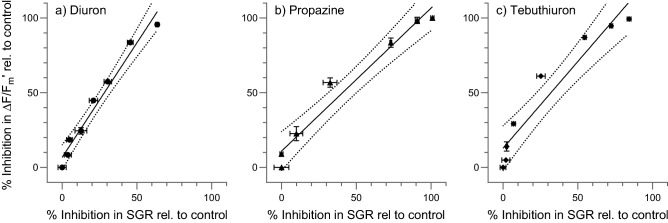
Linear regression model fits of the 24 h effective quantum yield (ΔF/F_m_′) vs 3-day specific growth rate (SGR) inhibition (solid black line) and 95% confidence interval (black dashed line) for (**a**) Diuron; (**b**) Propazine; (**c**) Tebuthiuron (mean ± SD; n = 5 per treatment).

A comparison of ΔF/F_m_′ and SGR inhibition due to PSII herbicides demonstrated that inhibition of ΔF/F_m_′ was a more sensitive endpoint than inhibition of SGR (Fig. 3, Table 3). The regression analyses indicated linear relationships between response types for all three PSII herbicides with slopes that were close to unity (Table 3). However, the comparison of the EC_50_ ratios for SGR : ΔF/F_m_′, which ranged from 2.0 to 3.9 (Table 2), revealed that inhibition in ΔF/F_m_′ was on average a threefold more sensitive endpoint than inhibition in growth.

**Table 3:** Linear regression coefficients of the effective quantum yield (ΔF/F_m_′) vs specific growth rate (SGR) for Diuron; Propazine; Tebuthiuron.

Herbicide	Slope (95% confidence interval)	R^2^
Diuron	1.53 (1.26–1.80)	0.97
Propazine	0.96 (0.74–1.17)	0.96
Tebuthiuron	0.92 (0.82–1.50)	0.92

## Discussion

### Toxicity of PSII herbicides to microalgae

The three PSII herbicides induced substantial reductions in both ΔF/F_m_′ and SGR of *C. muelleri* at relatively low concentrations (Table 2). PSII herbicides exert their toxicity by inhibiting the electron transport in the PSII complex, resulting in both reduced production and damage to the PSII system due to light-induced oxidative stress caused by formation of reactive oxygen species in the reaction center itself^48^. Prolonged exposure to reactive oxygen species can cause irreversible cell damage ultimately leading to cell death^49^. The inhibition of ΔF/F_m_′ in *C. muelleri* by PSII herbicides can indicate both reduced photosynthetic efficiency caused by blockage of electron transport driving production and damage to PSII and both mechanisms are likely to have contributed to the inhibition in SGR. Based on the EC_50_s in this study, the phenylurea herbicide diuron was the most toxic herbicide towards *C. muelleri*, while the triazine herbicide propazine and the phenylurea herbicide tebuthiuron were 8–15-times less toxic to ΔF/F_m_′ and SGR of *C. muelleri* (Table 2). Although PSII herbicides share the same mode of action toxicities of these herbicides significantly differed even for herbicides within the same chemical class, as demonstrated here for the phenylurea herbicides. Toxicity differences between herbicides of the same mode of action are consistent with other reports for marine microalgae^26,30^. The physico-chemical properties of the herbicides differ (Table S-1) but there was no clear direct relationship between these properties (K_OW_, water solubility etc.) and their toxicities. It is likely that herbicides that have greater affinities to the Q_B_ binding site and faster binding rates have greater toxic potential^50^.

Diuron is the most widely studied PSII herbicide with respect to tropical marine species, including corals^51^, foraminifera^52^, and macroalgae^53^ and inhibition of ΔF/F_m_′ is the most commonly reported toxic endpoint. However, ecologically relevant endpoints related to mortality, reproductive effects and growth inhibition are required for WQGV derivation^28^, and growth inhibition is more often reported for marine microalgae. For example, 3–7-day SGR inhibition EC_50_ values range between 0.55–110 μg L^−1^ diuron across 27 marine microalgal species (Table 4)^35,54^. The 3-day SGR EC_50_ value derived here for *C. muelleri* places this species among the more sensitive of microalgal species in this dataset, but direct comparisons among toxicity tests should be made with caution due to differences in experimental conditions. However, direct comparisons of EC_50_s against the equivalent thresholds of the marine cryptophyte *Rhodomonas salina* is valid as this species was tested in the same laboratory under identical test conditions^26^. This comparison revealed that *C. muelleri* was twofold less sensitive to diuron. The toxic effects of the alternative PSII herbicide propazine on marine microalgae have been investigated in only two other studies. The SGR EC_50_ value for *C. muelleri* (Table 4) indicated this species was around fourfold less sensitive than the diatom *Skeletonema costatum* (Table 4), but unlike diuron twice as sensitive to propazine compared to *R. salina* (Table 4). Although tebuthiuron is considered a priority herbicide, little data has been published on its toxic effects on marine microalgae. Indeed, chronic toxicity values (EC_50_) were only reported for the marine diatom *S. costatum* (Table 4) and *R. salina* which was almost 2-times more sensitive (Table 4) than the reported EC_50_ value for *C. muelleri* (Table 4).

**Table 4:** Toxicity values for *Chaetoceros muelleri* and other marine microalgae.

Herbicide	Phyla	Species	Duration	Endpoint SGR (µg L^-1^)		Endpoint ΔF/F_m_′ (µg L^-1^)		Reference
				EC_10_	EC_50_	EC_10_	EC_50_	
Diuron	Bacillariophyta	***Chaetoceros muelleri***	**3 d; 24 h**	**1.8**	**12**	**0.97**	**4.3**	**Present study**
		*Navicula* sp.	3 d	2.3	7.7	1.0	5.6	Magnusson et al*.*^30^
		*Navicula* sp.	4 h			0.78	2.6	Magnusson et al*.*^30^
		*Phaeodactylum tricornutum*	4 h			0.42	2.7	Magnusson et al*.*^30^
		*Cylindrotheca closterium*	4 h			0.63	4.4	Magnusson et al*.*^30^
		*Thalassiosira pseudonana*	4 d	1.6	4.3			Bao et al*.*^55^
		*Skeletonema costatum*	4 d	3.8	5.9			Bao et al.^55^
		*Navicula forcipata*	4 d		27			Gatidou and Thomaidis^56^
		*Phaeodactylum tricornutum*	2 h			0.84	18	Muller et al*.*^42^
		*Nitzschia pungens*	4 d		6.6			Jung et al.^57^
		*Chaetoceros gracilis*	3 d		36			Koutsaftis and Aoyama^58^
		*Phaeodactylum tricornutum*	7 min			0.11		Bengtson Nash et al*.*^43^
		*Nitzschia closterium*	14 min			0.10		Bengtson Nash et al*.*^43^
		*Phaeodactylum tricornutum*	4.5 h				1.8	Sjollema et al*.*^41^
		*Thalassiosira pseudonana*	4.5 h				2.9	Sjollema et al*.*^41^
	Cryptophyta	*Rhodomonas salina*	3 d; 24 h	2.7	13	0.60	3.0	Thomas et al*.*^26^
	Chlorophyta	*Nephroselmis pyriformis*	3 d	5.1	7.7	1.1	5.8	Magnusson et al*.*^30^
		*Nephroselmis pyriformis*	4 h			0.32	2.06	Magnusson et al*.*^30^
		*Dunaliella tertiolecta*	> 45 min			0.11		Bengtson Nash et al*.*^43^
		*Dunaliella tertiolecta*	4.5 h				2.9	Booij et al*.*^59^
		*Dunaliella tertiolecta*	4 d		9.2			DeLorenzo et al.^60^
	Cyanobacteria	*Chroococcus minor*	7 d	0.44	4.7			Bao et al*.*^55^
		*Synechococcus* sp.	4 d	12	110			Bao et al*.*^55^
		*Synechococcus* sp*.*	3 d		0.55			Devilla et al*.*^61^
	Haptophyta	*Coccolithus huxleyi*	3 d		2.3			Devilla et al*.*^61^
	Dinoflagellata	*Symbiodinium* sp.	10 h				2.3	Jones and Kerswell^51^
		*Symbiodinium* sp.	24 h			0.64	1.4	Mercurio et al.^62^
		*Dunaliella sp.*				1.0	4.4	Mercurio et al.^62^
Tebuthiuron	Bacillariophyta	***Chaetoceros muelleri***	**3 d; 24 h**	**27**	**187**	**7.0**	**48**	**Present study**
		*Skeletonema costatum*	5 d		60			USEPA^54^
		*Navicula* sp*.*	4 h			17	94	Magnusson et al*.*^30^
		*Phaeodactylum tricornutum*	4 h			7.6	51	Magnusson et al*.*^30^
		*Cylindrotheca closteriuma*	4 h			10	77	Magnusson et al*.*^30^
	Chlorophyta	*Nephroselmis pyriformis*	4 h			2.3	12	Magnusson et al*.*^30^
	Cryptophyta	*Rhodomonas salina*	3 d; 24 h	28	112	2.7	16	Thomas et al*.*^26^
	Dinoflagellata	*Symbiodinium* sp.	10 h				175	Jones and Kerswell^51^
Propazine	Bacillariophyta	***Chaetoceros muelleri***	**3 d; 24 h**	**22**	**98**	**8.1**	**49**	**Present study**
		*Skeletonema costatum*	5 d		25			USEPA^54^
	Cryptophyta	*Rhodomonas salina*	3 d; 24 h	42	188	5.9	40	Thomas et al*.*^26^
Haloxyfop	Bacillariophyta	***Chaetoceros muelleri***	**3 d; 24 h**	**> 4570**	**> 4570**	**> 4570**	**> 4570**	**Present study**
	Cryptophyta	*Rhodomonas salina*	3 d; 24 h	> 3700	> 3700	> 3700	> 3700	Thomas et al*.*^26^

Herbicide toxicity to marine microalgae including data from the USEPA ECOTOX Database^54^ and other publications using similar methods as those used in the present study (i.e. experimental conditions, ecological endpoint). Rows in bold indicate results from the present study.

Differential responses of microalgae to PSII herbicides may partially be due to related differences in the molecular architecture of the D1 protein, as well as different mechanisms of photosynthetic acclimation to light^63^. For example, chlorophytes are often reported to be more susceptible to herbicides compared to ochrophytes due to differences in light-harvesting pigments and adaptation of ochrophytes to low light conditions^64–66^. Compared to chlorophytes, diatoms such as *C. muelleri* may apply an extra carbon fixation pathway, for example β-carboxylation that could compensate for herbicide-induced reduction in PSII-based photosynthesis, allowing some metabolism to continue^67,68^. Community changes of microalgae in response to chronic PSII herbicide exposure have been observed in several studies. For example, pollution-induced community tolerance in tropical estuarine periphyton in response to chronic diuron exposures was observed by Magnusson et al.^69^, leading to a shift in species composition towards communities dominated by diatoms. The mechanisms leading to community tolerance in microalgae were not fully investigated but may be related to the ability in some diatom species to switch to heterotrophic nutrient acquisition under these conditions^40,70^. These clear but often unpredictable differences in responses to herbicide exposure between alga stresses the importance of incorporating microalgae species from different taxa when deriving SSDs and WQGVs for environmental protection purposes.

### Ecological risk of PSII herbicides

There are current marine WQGVs for diuron and tebuthiuron, but not for propazine in marine or freshwater environments^27^. Updated guideline values have recently been proposed (PGVs) for all three herbicides, however there were not enough data for marine phototrophs available to develop WQGVs for tebuthiuron and propazine, and therefore were derived from SSDs based on toxicity thresholds from both marine and freshwater taxa^35,36^. In fact, only two of seven species in the tebuthiuron SSD were marine and one of five species in the propazine SSD, highlighting the lack of tropical marine toxicity data for these herbicides. Consequently, the modeled distribution of these data resulted in low- to moderate-reliability PGVs that may not represent adequate protection to marine microalgae. Very high-reliability PGVs were able to be derived only for diuron as sufficient chronic toxicity data for marine phototrophs (in total 20 species) were available^35^.

Diuron, propazine, and tebuthiuron are all approved and registered for use in agricultural industries in the GBR catchment area^71^ but are more tightly regulated in the US^72^ and tebuthiuron and propazine do not have regulatory approval within the European Union^73^. From the most recent water quality monitoring year (2017–2018), these herbicides were reported as among the most frequently detected and abundant herbicides in nearshore waters of the GBR^24^ with frequencies between 40–80% in fixed (long-term) monitoring sites using passive samplers^24^. Maximum concentrations of these herbicides (typically identified in the Mackay-Whitsunday region) ranged from < 5 ng L^−1^ tebuthiuron and propazine to 778 ng L^−1^ diuron^24^. The concentration estimates from passive samplers can accurately estimate month-long averages, but concentrations of individual herbicides can reach over threefold higher concentrations during shorter duration pulses^74^. The 99% species protection (PC99) PGV of diuron (0.43 µg L^−1^), propazine (2.2 µg L^−1^), and tebuthiuron (4.7 µg L^−1^)^35,36^ were lower than the NEC values (1.47, 12.9 and 16 µg L^−1^, respectively) derived in this study, indicating that *C. muelleri* would be protected by the PGVs and are unlikely to be affected by most GBR field exposure concentrations of these herbicides individually. However, these highly mobile PSII herbicides^47^ have very long half-lives in marine waters^75^, contributing to their frequent year-round detection in complex mixtures^24,29^. It is therefore important that the risks posed by PSII herbicides should not be assessed individually. Instead, individual contributions to the risk posed by multi-herbicide mixtures should be assessed using ms-PAF^33^ which accounts for all herbicides that have reliable SSDs (and WQGVs). The individual toxicity thresholds (i.e. NECs) identified for *C. muelleri* here are intended to contribute to the future derivation of high-reliability marine WQGVs for the PSII herbicide diuron, propazine, and tebuthiuron and support assessments of cumulative risks of herbicide mixtures using ms-PAF.

### Toxicity of non-PSII herbicides on microalgae

Haloxyfop belongs to the family of phenoxy herbicides and has been developed as a selective herbicide that is mainly absorbed through the foliage and roots of plants with subsequent hydrolysis to the acid, which is herbicidally active^76^. Haloxyfop inhibits the acetyl-CoA carboxylase (ACCase) enzyme that is involved in the synthesis of fatty acids^76^ and exists in two forms, the multi-subunit, prokaryotic (heteromeric) form and the multi-domain, eukaryotic (homomeric) form and in two locations (cytosol and plastid)^77^. Haloxyfop and other ACCase inhibitors target primarily the eukaryotic form of the enzyme rather than the prokaryotic form^78^. In the present study, both, SGR and ΔF/F_m_′ of *C. muelleri* were nonresponsive to haloxyfop after 3-day exposure at the maximum concentration of 4570 µg L^−1^. In plants, both forms of ACCase enzyme have been described; however, some studies have indicated that certain microalgae, including some rhodophytes and chlorophytes, only contain the prokaryotic ACCase enzyme in their plastids^77,79^, possibly explaining the insensitivity of *C. muelleri* towards haloxyfop. There is only one other study on the toxicity of haloxyfop to marine phototrophs which reported a similar insensitivity in the marine cryptophyte *Rhodomonas salina*, with no inhibition of ΔF/F_m_′ or SGR at the highest concentration of 3700 µg L^−1^ (Table 4). Additionally, there were chronic toxicity data for one freshwater chlorophyte, *Scenedesmus subspicatus,* which reported 4-day no observed effect level (NOEL) and EC_50_ (biomass yield, growth rate, area under the growth curve) values of 5000 μg L^−1^ and 106,000 μg L^−1^ , respectively^54^.

### Ecological risk of haloxyfop

Haloxyfop has only recently been included in monitoring programs in GBR waters and detection frequencies (< 33%) and concentrations measured by passive sampling are generally low (< 1 ng L^−1^) in marine waters^24^. There are no current WQGVs for haloxyfop in freshwater or marine environments^27^, while the PGVs are based on toxicity data of a combination of one freshwater phototroph and five marine and freshwater heterotrophs^36^. However, the modelled distribution of these data indicated a poor data fit and subsequently resulted in low-reliability PGVs^36^. The PC99 PGV of 590 µg L^−1^ is an order of magnitude lower than the NEC value we report for *C. muelleri* of > 4570 µg L^−1^, indicating *C. muelleri* is well protected by this PGV and that environmental concentrations currently recorded do not pose a risk to this species in comparison to PSII herbicides. It should further be noted that haloxyfop may be less bioavailable in seawater due to its molecular structure. Haloxyfop contains a carboxyl group (COOH) which can result in complexation with Mg^2+^ and Ca^2+^ ions in seawater^80^, or stabilize the herbicide at the seawater:air interface^81^. These chemical properties could reduce the exposure and bioavailability of haloxyfop to marine species accounting for the low toxicities reported for the marine microalgae *Rhodomonas salina*^26^ and *C. muelleri*. Nevertheless, the acute and chronic toxicity data presented here will contribute towards deriving more reliable marine WQGVs for haloxyfop in the future, enabling the contribution of haloxyfop to the total herbicide risk to be assessed using ms-PAF.

### Relationship between inhibition of effective quantum yield and growth

SSDs are currently developed using toxicity data from chronic exposure experiments, and ecologically relevant endpoints, such as inhibition of growth are preferred^28^. However, several studies have recommended the use of PAM fluorometry for estimating adverse biological effects of PSII herbicides^26,40,82–84^. In this study, the NEC and EC_x_ values derived for SGR inhibition were all consistently higher than the respective NEC and EC_x_ values estimated for inhibition in ΔF/F_m_′ (Table 2). In fact, the direct comparison between EC_50_ values of each PSII herbicide calculated for SGR and ΔF/F_m_′ inhibition revealed that SGR was on average 3-times less sensitive to PSII herbicide exposures than ΔF/F_m_′ (Table 2). The relationship between herbicide inhibition of SGR and ΔF/F_m_′ for marine microalgae has only been investigated in two earlier studies. Thomas et al.^26^ similarly reported that the SGR of the cryptophyte *R. salina* was on average 4-times less sensitive to PSII herbicide exposure than the photoinhibition endpoint. In a study by Magnusson et al*.*^40^ the relationship between SGR and ΔF/F_m_′ inhibition by PSII herbicides was closer to 1:1 for two tropical benthic microalgae; *Navicula* sp. and *Nephroselmis pyriformis*. However, it is not necessarily expected that the reduced electron transport, due to the binding of PSII herbicides to the D1 protein is directly linked (1:1) to reduced growth rates for all taxa and experimental conditions. ΔF/F_m_′ values are affected by actinic (ambient) light intensity and acclimation period of the test species and this in turn can affect the sensitvity of ΔF/F_m_′ inhibition as an ecotoxicological endpoint^85^. Furthermore, the complex relationship between light-driven productivity and nutrient availability as well as species-specific physiologies make direct comparisons with prior studies more difficult. Nevertheless, the consistency of the linear relationship between toxicity thresholds based on ΔF/F_m_′ and SGR for *C. muelleri* and three other marine species^26,40^ clearly demonstrated that inhibition of ΔF/F_m_′ in PSII is directly linked to reduced growth in marine microalgae. This highlights the applicability of fluorescence microplate toxicity assays to quantify sub-lethal effects of PSII herbicides on microalgae. Indeed, the strength and consistency of this relationship, as well as the clear mechanistic link between inhibition of ΔF/F_m_′ and growth rates indicates that for microalgae, inhibition of ΔF/F_m_′ should be considered a valid bioindicator of ecological relevance and moreover, that chronic ΔF/F_m_′ toxicity endpoints could contribute to deriving WQGVs for PSII herbicides in the future.

## Conclusion

Although a revision of the current WQGVs has recently been proposed, most of the PGVs were derived from freshwater toxicity thresholds and consequently are of low reliability, signifying data gaps for tropical marine species, especially for marine phototrophs. Here, we demonstrated that exposures of the diatom *C. muelleri* towards PSII herbicides resulted in substantial reductions of ΔF/F_m_′ within 24 h, which subsequently inhibited growth rates over 3-day chronic exposures. Inhibition in ΔF/F_m_′ was on average 3-times more sensitive than inhibition in growth to PSII herbicide exposure, but was linearly related, highlighting the applicability of fluorescence microplate toxicity assays to quantify sub-lethal impacts of PSII herbicides on microalgae. These results are consistent with the responses of three other microalgal species^26,40^, supporting the notion that inhibition of ΔF/F_m_′ could be considered a valid bioindicator of ecological relevance and moreover, that chronic ΔF/F_m_′ toxicity endpoints could contribute to deriving future WQGVs for PSII herbicides. In contrast, the non-PSII herbicide haloxyfop did not affect SGR and ΔF/F_m_′ in *C. muelleri* at very high concentrations, suggesting haloxyfop pose little risk to this microalga in the marine environment. While the toxicity thresholds (NECs and EC_10_s) derived here were all higher than concentrations detected in GBR monitoring programs, high-reliability WQGVs that underpin their regulation are generally lacking, especially for alternative herbicides. The toxicity thresholds (i.e. NECs) identified here for *C. muelleri* are therefore valuable contributions to the future derivation of high-reliability marine WQGVs for the PSII herbicide diuron, propazine, and tebuthiuron as well as the non-PSII herbicide haloxyfop, supporting improvements in cumulative risk assessments of herbicide mixtures using ms-PAF.

## Methods

### Diatom cultivation

The diatom *Chaetoceros muelleri*^86^ (strain CS-176) was purchased from the Australian National Algae Supply Service, Hobart. The genus *Chaetoceros* is considered as one of the most diverse genera of diatoms in the marine phytoplankton with a global distribution ranging from temperate to tropical regions^87^. Besides its importance as primary producer, this brackish-marine diatom is commonly used in aquaculture hatcheries for its high lipid content^88^. Prior to experimentation, cultures of *C. muelleri* were acclimatized under experimental conditions (below) for a period of two weeks and maintained in 500 mL Erlenmeyer flasks as batch cultures in exponential growth phase with weekly transfers of 70 mL algae suspension into 350 mL sterile culture medium. The culture medium was prepared from sterile 0.5 µm filtered seawater (FSW; pH 8.0, salinity 35.0 psu) enriched with Guillard’s f/2 marine medium^89^ (0.5 mL of AlgaBoost F/2, AusAqua in 1 L 0.5 µm-FSW). Cultures were continuously aerated and kept at 27.0 ± 1 °C and 35 psu. Cultures were exposed to a 12:12 h light:dark cycle with light supplied from two fluorescent tubes (Osram Lumilux Cool White 36 W) and irradiance adjusted to 100–110 μmol photons m^–2^ s^–1^.

### Preparation of test solutions

Herbicides to be tested in this study were selected based on their application and detection rate in GBR monitoring programs and those currently lacking marine water quality guideline values. Diuron was chosen as a reference toxicant as its toxicity to a wide variety of microalgae is well studied^26,40^. Toxicant stock solutions were prepared using PESTANAL analytical grade products (Sigma-Aldrich, HPLC ≥ 98% purity): diuron (CAS 330-54-1), propazine (CAS 139-40-2), tebuthiuron (CAS 34014-18-1), haloxyfop (CAS 72619-32-0). Stock solutions of diuron (10 mg L^−1^), propazine (8.5 mg L^−1^), tebuthiuron (50 mg L^−1^), and haloxyfop (40 mg L^−1^) were prepared in sterile 500 mL Schott glass bottles using Milli-Q water or FSW and sonicated for a minimum of 2 h. A solvent carrier was used for the preparation of the diuron stock (HPLC-grade ethanol (< 0.001% (v/v) in exposure). No solvent carrier was used for tebuthiuron, propazine and haloxyfop.

### Toxicity testing procedure

Chronic toxic effects of herbicides on the specific growth rate (SGR) of *C. muelleri* were tested in 72 h static exposure experiments according to the test procedure by Thomas et al.^26^ and based on OECD Test No. 201^46^. Initially, 15 mL of algae inoculum was taken from 4-day-old *C. muelleri* culture (approximately 2 × 10^6^ cells mL^−1^) in exponential growth phase and washed with 15 mL sterile FSW by centrifugation in 50 mL falcon tubes at 1500×*g* for 5 min (Eppendorf Centrifuge 5810 R, Bio-strategy). The supernatant was decanted, and the remaining algae pellet homogenized in 30 mL FSW by vortexing. The centrifugation process was repeated three times prior to the start of each toxicity test. After the final washing, the cell pellet was re-suspended in 15 mL of sterile 0.5 µm-FSW and the cell density of the concentrated algae suspension was measured from two 500 µL sub-samples by flow cytometry. The desired inoculum was calculated to have a starting cell density of 3 × 10^3^ cells mL^−1^ in the toxicity tests. Individual *C. muelleri* working suspensions for each herbicide treatment were prepared in 100 mL Schott glass bottles by adding the required algae inoculum and sterile 0.5 µm-FSW. Each Schott glass bottle was finally dosed with a range of herbicide concentrations (Table S-2). Five replicated aliquots of 10 mL were transferred from the individual 100 mL Schott glass bottles into sterile 20 mL glass scintillation vials and incubated at 27.5 ± 0.4 °C under a 12:12 h light:dark cycle at 90–100 μmol photons m^–2^ s^–1^ (Osram Lumilux Cool White 36 W). Vials were randomized and swirled daily. Bioassays for each herbicide were performed on different days with fresh algae, FSW and herbicide stocks. In each bioassay, a control (no herbicide) and reference (diuron, 4 µg L^−1^) treatment were included to indicate test consistency.

### Cell density measurements

Sub-samples of 500 µL were taken from each replicate to measure cell densities of algal populations at 0 h and 72 h using a flow cytometer (BD Accuri C6, BD Biosciences, CA, USA) equipped with red and blue lasers (14.7 mW 640 nm Diode Red Laser 20 mW 488 nm Solid State Blue Laser) and standard filter setup^26^. The flow rate was set to 35 µL min^−1^, 16-µm core size with a sample volume of 50 µL. Cell densities were obtained by plotting a two-dimensional cytogram. A fixed gating was used around the viable (chlorophyll fluorescing) cells, which allowed for differentiation of non-algal particles (debris) and dead cells from viable cells, which typically represented 80–95% of particles counted (control treatment at 72 h). Aliquots were run in duplicates and an average taken of the number of events that occurred within the gated region. This process was then repeated for each replicate per treatment. Specific growth rates (SGR) were expressed as the logarithmic increase in cell density from day i (t_i_) to day j (t_j_) as per Eq. (1), where SGR_i-j_ is the specific growth rate from time i to j; X_j_ is the cell density at day j and X_i_ is the cell density at day i^46^:

1 $${\text{SGR}}_{\text{i-j }}\text{ = }\frac{{\text{ln X}}_{\text{j }}- \text{ } {\text{ln X}}_{\text{i}}}{{\text{t}}_{\text{j}}\text{ - }{\text{t}}_{\text{i}}} \, {\text{(day}}^{-1}\text{)}$$

SGR relative to the control treatment was used to derive chronic effect values (EC_10_ and EC_50_) and no effect concentrations (NEC) for growth inhibition. A test was considered valid if the mean SGR of control replicates was ≥ 0.92 day^−1^, the percent coefficient of variation (% CV) of the average specific growth rate of control cultures did not exceed 10% and the pH of the control medium did not increase by more than 1-unit during the test^46^.

### Chlorophyll fluorescence measurements

The effects of herbicide on chlorophyll fluorescence were measured as effective quantum yield (ΔF/F_m_′) using imaging PAM fluorometry (I-PAM, Walz, Germany)^83,90^ following a single 12:12 h light:dark cycle (90–100 μmol photons m^–2^ s^–1^)^26^. Light-adapted minimum fluorescence (F) and maximum fluorescence measurements (F_m_′) were taken in 48-well plates (Nunclon Delta, Thermo Scientific) from which the effective quantum yield was calculated as per Eq. (2) ^90^. An initial cell density of approximately 1 × 10^6^ cells mL^−1^ was used to obtain ΔF/F_m_′ measurements > 0.45 with the following I-PAM settings: actinic light = 1 (corresponding to photosynthetically active radiation (PAR) of 100–110 μmol photons m^-2^ s^-1^), measuring intensity = 9, gain = 1; damp = 2.

2 $$\frac{{\Delta}{\text{F}}}{{{\text{F}}{\text{m}}}^{{\prime}}}\text{ = }\frac{{\text{F}}{\text{m}}{^{\prime} - F}}{{\text{F}}{\text{m}}{^{\prime}}}$$

Prior to herbicide exposure a screening process of control treatments was performed to ensure consistent ΔF/F_m_′ measurements > 0.45. Diuron was used as a referent toxicant (4 µg L^−1^) to monitor inhibition response between replicated algae cultures.

### Chemical analyses

Physical and chemical characteristics of each treatment were measured at 0 h and 72 h including pH and salinity (LAQUAact-PC110 Meter, HORIBA Scientific) and dissolved oxygen (HQ30D Portable Meter, HACH). Temperature was logged in 10-min intervals over the total test duration (HOBO, Onset). Samples for chemical analysis were taken at start and end of herbicide exposure. Aliquots (1 mL) were transferred into 1.5 mL Liquid Chromatography amber glass vials and spiked with surrogate standards (i.e. diuron-D6, propazine-D6, and haloxyfop-D4) at a final concentration of 10 ng mL^−1^. Prior to analysis samples were stored at − 20 °C, defrosted and centrifuged. Herbicide concentrations were determined by HPLC–MS/MS using an SCIEX Triple Quad 6500 QTRAP mass spectrometer (SCIEX, Concord, Ontario, Canada) equipped with a TurboIonSpray probe^22,23^. The mass spectrometer was coupled to a Shimadzu Nexera X2 uHPLC system (Shimadzu Corp., Kyoto, Japan) using a Phenomenex Kinetex Biphenyl column (2.6 μm 50 × 2.1 mm 100 Å) for analyte separation. 5μL of sample was injected on to the column followed by a linear gradient starting at 10% B for 0.5 min, ramped to 100% B in 4.7 min then held at 100% for 4.0 min followed by equilibration at 10% B for 3.0 min (A = 1% methanol in Milli-Q water, B = 95% methanol in Milli-Q water, both containing 0.1% acetic acid). The mass spectrometer was operated in both positive and negative ion mode using a scheduled multiple reaction-monitoring method (sMRM). Positive samples were confirmed by retention time and by comparing transition intensity ratios between the sample and an appropriate calibration standard from the same run. The measured concentrations used for concentration–response modelling were derived from the geometric mean of measured start and end concentrations (time weighted average)^26^.

### Data analysis

Statistical analyses and threshold estimates were based on measured herbicide concentrations (Table S-1). The inhibition of SGR and ΔF/F_m_′ in *C. muelleri* by herbicides was quantified as per Eq. (3)^46^, where X_control_ is the average SGR or ΔF/F_m_′ of control and X_treatment_ is the average SGR or ΔF/F_m_′ of single treatments.

3 $$\text{\% Inhibition = }\frac{{\text{X}}_{\text{control}} - {\text{X}}_{\text{treatment}}}{{\text{X}}_{\text{control}}}\text{ x 100}$$

Concentrations that effectively inhibited SGR and ΔF/F_m_′ by 10% or 50% (EC_10_ and EC_50_) and their 95% confidence intervals relative to the control treatment were calculated from nonlinear regression (Sigmoidal, 4-parameter) using GraphPad Prism V 8.0.

The relative potencies of each herbicides was determined using the relative equivalent potencies (ReP) compared to the reference herbicide diuron (EC_50_ diuron/EC_50_ herbicide)^40^. ReP values > 1 indicate potencies proportionally greater than diuron and ReP values < 1 indicate potencies less than diuron. SGR and ΔF/F_m_′ data from haloxyfop experiments were analyzed using one-way analysis of variance (ANOVA) to determine any significant differences between treatments for each endpoint.

The estimations of no effect concentrations (NEC) that have no adverse effect on a species were calculated in R (Version 3.6.1) as per Thomas et al.^26^. Proportional decline in SGR (1-inhibition) was modelled as a function of log concentration of each herbicide using a Bayesian non-linear gaussian model using the R package jagsNEC^91^. This model has been specifically developed to derive no effect concentrations (NECs) and is defined by Eq. (4) ^92^:

4 $$\text{E }\left[{\text{Y}}_{\text{i}}|{\text{x}}_{\text{i}}\right]\text{=}{\mu}_{\text{i}}{= \alpha} {\text{exp}}\left[{- \beta}\left({\text{x}}_{\text{i}}{- \gamma}\right)\text{I}\left({\text{x}}_{\text{i}}{- \gamma}\right)\right]{- \Delta}$$

$\text{E[}{\mathrm{Y}}_{\mathrm{i}}$|x_i_] is the mathematical expectation of Y_i_ (the response, e.g. in this case the proportional decline in SGR) conditional on a given concentration x_i_. The model parameters for the generalised case are $\alpha$ (the response at zero or low concentrations, also called ‘top’), $-\beta$ (the rate of decay in the response after the NEC) and γ (the NEC value)^92^. For a gaussian *Y*, as used here, the model has the additional parameters Δ (an offset or intercept) and σ (the random error variance in *Y*) (see Thomas et al.^26^ for further details).

## Supplementary information

Supplementary Information 1.
