# Supplementary material for: Toxicity of the herbicides diuron, propazine, tebuthiuron, and haloxyfop to the diatom Chaetoceros muelleri
Source: Sci Rep. 2020 Nov 11;10:19592. doi: 10.1038/s41598-020-76363-0 (PMC7658992; doi:10.1038/s41598-020-76363-0)
Supplement: Supplementary file 1 — Supplementary Information 1. [file 41598_2020_76363_MOESM1_ESM.pdf]

Toxicity of the herbicides diuron, propazine, tebuthiuron, and haloxyfop to the diatom *Chaetoceros muelleri*

Marie C. Thomas[1*], Florita Flores[1], Sarit Kaserzon[2], Timothy A. Reeks[2] & Andrew P. Negri[1]

[1]Australian Institute of Marine Science, Townsville, QLD 4810, Australia
[2]Queensland Alliance for Environmental Health Sciences (QAEHS), The University of Queensland, Woolloongabba, QLD 4102, Australia

[*]m.thomas@aims.gov.au

Table S-1. Summary of the chemical properties of herbicides used in this study. Data were compiled using the Pesticide Properties Database (https://sitem.herts.ac.uk/aeru/ppdb/)[47].

**Chemicals**	**Diuron**	**Propazine**	**Tebuthiuron**	**Haloxyfop**
CAS No.	330-54-1	139-40-2	34014-18-1	72619-32-0
Chemical class	Phenylurea	Triazine	Phenylurea	Phenoxy
Molecular weight (g mol^{-1})	233.09	229.71	228.31	375.70
Water Solubility (mg L^{-1}) at 20 °C	35.6	8.6	2500	7.9
Logarithm of the octanol-water partition coefficient (log Kow; pH 7 at 20 °C)	2.87	3.95	1.79	4.0
Logarithm of the organic carbon water partition coefficient (log Koc)	2.83	2.19	1.90	(not available)
Bio-concentration factor BCF (L kg^{-1})	9.45	62	2.6	17

Table S-2. Summary of stock solutions and nominal and measured concentrations used for cell density (Growth test) and chlorophyll fluorescence (PAM test) measurements. Stock solutions were prepared in Milli-Q water or filtered seawater (FSW). Measured concentrations are the average of initial and final concentration in each test. Nominal and measured concentrations in µg L^{-1}.

Herbicide	Stock solution (mg L^{-1})	FSW/Milli-Q	Nominal concentration		Measured concentration	
			Growth test	PAM test	Growth test	PAM test
Diuron	10	Milli-Q	0; 0.5; 1; 2; 4; 6; 10; 20	0; 0.5; 1; 2; 4; 6; 10; 20; 30	0; 0.51; 1.02; 2.04; 4.08; 6.12; 10.2; 20.4	0; 0.51; 1.02; 2.04; 4.08; 6.12; 10.2; 20.4; 30.6
Propazine	8.5	FSW	0; 10; 30; 100; 300; 1,000; 3,000	0; 1; 3; 10; 30; 100; 300; 1,000; 3,000	0; 6.17; 18.5; 61.7; 185.0; 617; 1,850	0; 0.62; 1.85; 6.17; 18.5; 61.7; 185.0; 617; 1,850
Tebuthiuron	50	Milli-Q	0; 1; 10; 30; 100; 300; 600; 1,000	0; 0.5; 1; 10; 30; 100; 300; 600; 1,000	0; 0.77; 7.69; 23.1; 76.9; 231; 769	0; 0.38; 0.77; 7.69; 23.1; 76.9; 231; 769
Haloxyfop	40	FSW	0; 5,000; 10,000; 20,000; 30,000; 40,000	0; 5,000; 10,000; 20,000; 30,000; 40,000	0; 572; 1,144; 2,287; 3,432; 4,575	0; 572; 1,144; 2,287; 3,432; 4,575

Table S-3. Summary of water quality measurements. Physicochemical measurements of each treatment and test measured at test initiation (0 h) and test finalization (72 h) including pH, salinity, and dissolved oxygen (DO). Temperature was logged in 10-min intervals over the total test duration (mean ± SD).

Herbicide	Nominal concentration	Test initiation (0 h)				Test finalization (72 h)				
		pH	Salinity	DO		pH	Salinity	DO		Temperature
	µg L^{-1}	units	psu	mg L^{-1}	%	units	psu	mg L^{-1}	%	°C
Diuron	0	8.49	35.0	8.25	98.6	8.47	35.1	8.54	96.2	27.7 ± 0.33
	0.5	8.49	35.1	8.26	98.8	8.50	35.3	8.71	98.0	
	1	8.48	35.1	8.19	98.0	8.52	35.4	8.45	95.2	
	2	8.45	35.2	8.18	98.0	8.46	35.4	8.38	94.6	
	4	8.45	34.9	8.20	98.4	8.33	35.1	8.21	92.7	
	6	8.48	35.1	8.22	98.5	8.29	35.4	8.21	92.7	
	10	8.43	35.1	8.18	98.0	8.25	35.4	8.24	93.2	
	20	8.43	35.2	8.17	97.9	8.17	35.4	8.09	91.8	
Propazine	0	8.18	33.1	8.51	97.0	8.27	33.2	8.68	98.8	27.8 ± 0.31
	10	8.25	33.2	8.42	95.8	8.39	33.4	8.66	98.4	
	30	8.27	33.2	8.39	95.5	8.33	33.6	8.47	96.4	
	100	8.19	33.2	8.40	95.7	8.27	33.5	8.17	93.0	
	300	8.17	33.4	8.36	95.2	8.21	33.5	8.08	92.2	
	1000	8.16	33.3	8.37	95.3	8.18	33.5	8.08	92.3	
	3000	8.18	33.3	8.40	95.6	8.23	33.6	8.02	91.7	
	[a]Diuron	8.17	33.3	8.33	95.0	8.23	33.5	8.22	94.1	

Tebuthiuron	0	8.00	34.5	8.51	97.0	8.36	34.8	7.89	90.5	26.9 ± 0.45
	1	8.07	34.3	8.42	95.8	8.40	35.3	8.06	92.1	
	10	8.09	34.4	8.39	95.5	8.41	35.3	7.92	90.6	
	30	8.10	34.2	8.40	95.7	8.38	35.4	8.08	92.5	
	100	8.12	34.2	8.36	95.2	8.25	35.4	8.01	91.8	
	300	8.13	34.2	8.37	95.3	8.17	35.2	8.00	91.6	
	600	8.13	34.3	8.40	95.6	8.13	35.3	7.98	91.2	
	1000	8.13	34.5	8.33	95.0	8.11	35.4	8.02	91.7	
	[a]Diuron	8.14	35.0	8.18	93.1	8.17	35.7	7.98	91.4	
Haloxyfop	0	8.06	35.3	8.23	98.7	8.21	35.2	8.62	99.3	27.5 ± 0.38
	5	8.08	35.3	8.15	94.6	8.24	35.2	8.38	96.6	
	10	8.06	35.4	8.12	94.2	8.22	35.3	8.36	96.7	
	20	8.04	35.3	8.24	93.1	8.21	35.3	8.42	97.0	
	30	8.01	35.3	8.20	92.7	8.18	35.3	8.40	96.9	
	40	7.99	35.3	8.18	92.2	8.16	35.4	8.40	96.8	
	[a]Diuron	8.06	35.4	7.89	91.4	8.14	35.5	8.04	93.0	

[a]Reference treatment (diuron; 4 µg L^{-1})
